# High-Efficiency Expression of TAT-bFGF Fusion Protein in *Escherichia coli* and the Effect on Hypertrophic Scar Tissue

**DOI:** 10.1371/journal.pone.0117448

**Published:** 2015-02-23

**Authors:** Xuechao Jia, Haishan Tian, Lu Tang, Long Zheng, Lulu Zheng, Ting Yang, Bingjie Yu, Zhitao Wang, Peng Lin, Xiaokun Li, Xiaojie Wang

**Affiliations:** Key Laboratory Biotechnology Pharmaceutical Engineering, Wenzhou Medical University, Chashan University Park, Wenzhou, 325035, China; University of Tennessee, UNITED STATES

## Abstract

**Background:**

Basic fibroblast growth factor (bFGF) is a member of the fibroblast growth factor family that has effects on wounding healing and neuro-protection. However, it is difficult to use bFGF to treat diseases that are separated by physiological barriers, such as the dermal barrier and blood brain barrier.

**Methodology/Principal Findings:**

To improve bFGF’s penetration ability, we fused the recombinant human fibroblast growth factor (rhbFGF) gene with TAT. We constructed a pET3c vector that contained the recombinant bFGF gene and successfully expressed this gene in the E. coli strain BL21 (DE3) pLsS. The fusion protein was purified using CM Sepharose FF and heparin affinity chromatography. The purity of the TAT-rhbFGF was greater than 95%, as detected by SDS-PAGE. An *in vitro* MTT trial revealed that the modified bFGF significantly promoted the proliferation of NIH3T3 cells. The cell penetration trial and the mouse skin penetration trial demonstrated that the fusion protein had certain penetration abilities. The animal experiments confirmed that TAT-rhbFGF was effective in the treatment of the hypertrophic scars.

**Conclusions/Significance:**

We have successfully expressed and purified a TAT-rhbFGF fusion protein in this study. Our results have shown that the fusion protein had a greater ability to penetrate the dermal skin layer. TAT-rhbFGF improved the physical appearance of hypertrophic scars. TAT-rhbFGF may be a potential fusion protein in the treatment of dermal disorders, including hypertrophic scar.

## Introduction

Basic fibroblast growth factor (bFGF) is a member of the fibroblast growth factor family, which is widely distributed throughout various human and animal cells. bFGF was first abstracted and purified by Gospodarowicz in 1974[1]. In 1986, Abraham cloned the cDNA sequence of human bFGF[2]. Since then, human bFGF has been under extensive experimentation for improvement, and in these works, bFGF has been expressed in various species, such as E. coli[3], Picher pastoris[4] and silkworms[5]. Eventually, a recombinant human fibroblast growth factor (rhbFGF) was made for clinic treatment. bFGF is well known for its role in the process of wound healing[6], neuro-protection[7], cell proliferation and apoptosis[8]. Specifically, bFGF has been shown to play a significant role in wound healing in which bFGF promotes faster healing and fewer scars[9].

Hypertrophic scars are a common clinical skin disorder. The development of hypertrophic scars usually occurs in darker skinned patients and is associated with the proliferation of fibroblasts and the excessive deposition of extracellular matrix (ECM)[10,11]. In the wound, the invasive proliferation of fibroblasts leads to excessive expression of collagen proteins and keloids. bFGFs are believed to alleviate scar formation in a rabbit ear model by decreasing the collagen expression[12]. Because bFGF is a high molecular weight protein, it is difficult to deliver bFGF to the dermal tissue except through direct injection. In this work, we utilized cell-penetrating peptides (CPPs) to help the protein penetrate the scar.

CPPs are vehicles for the intracellular and transdermal delivery of macromolecules, and several transporters of CPPs have been previously described[13,14]. The HIV trans-activator of transcription (TAT) peptide has 9 basic amino acids and is a type of high-efficiency CPP. The TAT fusion protein has been investigated and was confirmed to be efficient in transdermal administration[15,16]. Because the recombinant protein solution was difficult to adhere to the skin surface, we utilized carbomer gel to maintain the protein on the skin for an extended period of time. Carbomer is a high-molecular weight, water-soluble polymeric resin. Carbomer is widely used for auxiliary substances in drug development[17]. Therefore, we fused the TAT peptide with rhbFGF and manufactured the carbomer gel to evaluate its potential effect on hypertrophic scars.

## Materials and Methods

### Reagents

Restriction enzymes NdeΙ, EcoRΙ, T4 DNA Ligase, DNA polymerase, plasmid purification kit and agarose gel DNA extraction kit were purchased from Dalian Takara (Dalian, China). DH5α and BL21 (DE_3_) pLsS were obtained from the Key Laboratory of Zhejiang Province Biotechnology and Pharmaceutical Engineering. The bFGF antibody was purchased from Santa Cruz Biotechnology (Santa Cruz, CA, USA). Hematoxylin, eosin and the One Step TUNEL Apoptosis Assay Kit were purchased from Beyotime (Shanghai, China).

### Animals

BALB/c mice (n = 15, 20 g) and Japanese big-ear white rabbits (n = 6, 2–2.5 kg), were obtained from the Laboratory Animal Center of Wenzhou Medical University and were treated strictly in accordance with international ethical guidelines and the National Institutes of Health Guide Concerning the Care and Use of Laboratory Animals. The experiments were carried out with the approval of the Animal Experimentation Ethics Committee of Wenzhou Medical University.

### Expression and Purification of TAT-rhbFGF

**Construction of TAT-rhbFGF expression vector.** The coding sequence of rhbFGF was obtained from a pET3c vector containing the sequence of recombinant human basic fibroblast growth factor (rhbFGF). Two forward primers containing the coding sequence of the transactivator of transcription protein transduction domain were used to fuse the TAT_49–57_ coding sequence with rhbFGF. The primers used to recombine and amplify the TAT_49–57_-rhbFGF were the following: forward primers: F1-5’-CGC CAT ATG CGC AAA AAA CGT CGT CAGC-3’, F2-5’-ACGTCGTCAGCGTCGCCGTCCAGCTTTGC-3’; reverse primer: R-5’-CCGGAATTCTTAGCTCTTAGCAGACATTGG-3’. The forward primer F1 and the reverse primer contained NdeΙ and EcoRΙ, respectively. To fuse the TAT_49–57_ coding sequence to rhbFGF, we first amplified a partial TAT sequence and a complete rhbFGF sequence with primers F2 and R. Primers F1 and R were then used to amplify the product obtained from the previous step to acquire the TAT-rhbFGF coding sequence. PCR was conducted with a 50 μl reaction mixture containing 0.5 μl PrimeSTAR HS DNA Polymerase (2.5 U/μl), 10 μl 5x PS buffer (Mg^2+^ plus), 4 μl dNTPs (2.5 mM each), 1 μl of both the forward and the reverse primers (10 μM each), 0.0625 μl pET3c-rhbFGF, and 33.5 μl ultra-pure water. The thermo-cycling parameters used for the PCR were the following: 10 s at 98°C for denaturation, 15 s at 61°C for annealing, and 1 min at 72°C for extension. After 28 cycles, the final product generated by primers F1 and R was digested with NdeΙ and EcoRΙ and was then ligated into the previously digested pET3c expression vector to create the pET3c-TAT-rhbFGF construct. The construct was transformed into E. coli DH5α. The accurate insertion of the gene into the plasmid was confirmed by automated DNA sequencing. After being amplified in E. coli DH5α, the expression vector was extracted and transformed into competent cells of E. coli strain BL21 (DE3) PlysS.

**Production and screening of TAT-rhbFGF.** To obtain the best expression strains of TAT-rhbFGF, the recombinant E. coli BL21 (DE3) PlysS with the correct TAT-rhbFGF sequence was shaken and cultured at 37°C and 200 rpm/min in 5 ml Luria-Bertani (LB) medium containing 100 μg/ml ampicillin. When the cell density reached an OD_600_ of 0.6, the cells were diluted with a final concentration of 1 mM IPTG as an inducer. After adding the inducer, we continued to incubate the cells at 37°C for 4 h with shaking at 200 rpm/min. The expression of each culture was analyzed using Coomassie brilliant blue staining of 15%(ν/ν) sodium dodecyl sulfate polyacrylamide gel electrophoresis (SDS-PAGE), and the expression level of the TAT-rhbFGF fusion protein was determined by densitometry. The greatest expression of the fusion protein transformant was reserved and used for subsequent experiments.

We then investigated the relationship of the IPTG concentration, the temperature, and the time after introduction with the expression of the fusion protein. After the best parameters were confirmed, we amplified and incubated the transformant in 500 ml LB medium. The bacteria were harvested by centrifugation at 8000 rpm for 10 min at 4°C. The cells were then resuspended in 20 mM Tris-HCl (pH 8.0) buffer containing 0.1 M NaCl and 10 mM ethylene diamine tetraacetic acid (EDTA). Subsequently, the cells were lysed by sonication for 20 min in an ice bath. The lysates were the centrifuged at 20000 rpm and 4°C for 30 min. Finally, the supernatant was transferred to a fresh tube and used for subsequent purification.

**Purification and identification of TAT-rhbFGF.** Considering the isoelectric point of the target protein, CM Sepharose Fast Flow was chosen for the purification of TAT-rhbFGF. Because the target protein had an affinity with heparin, we selected a heparin sepharose column for further purification The CM column was equilibrated with 200 ml equilibrium liquid (20 mM PB, 0.1 M NaCl, pH 7.0) at a rate of 2 ml/min. Subsequently, the supernatant was applied to the column at a rate of 1.5 ml/min. After the target protein was bound to the column, we washed the column with 50 ml equilibrium liquid at a flow rate of 2 ml/min and then washed with elution buffer (20 mM PB, 0.6 M NaCl, pH 7.0). The elution liquid was subsequently bound to the heparin sepharose column that was previous equilibrated using equilibrium buffer (20 mM PB, 0.6 M NaCl, pH 7.0). After binding to the column, we re-equilibrated the column and eluted with elution buffer (20 mM PB, 1.2 M NaCl, pH 7.0). The purity of the fusion protein was assessed using SDS-PAGE, and the concentration was determined using the Bradford method. The immune reactivity of the target protein was verified by western blotting, and the purified protein was subpackaged and reserved at -70°C.

**Analysis of the mitogenic activity of TAT-rhbFGF.** The biological activity of the TAT-rhbFGF was assessed by its ability to accelerate proliferation in the NIH 3T3 cell line (American Type Culture Collection, Rockville, MD). The cells were transferred into a 96-well plate (7×10^3^ cells per well) and were incubated in DMEM supplemented with 10% fetal bovine serum (FBS), 100 U/ml ampicillin, and 100 U/ml streptomycin for 12 h at 37°C with 5% CO_2_. The medium was then replaced by DMEM containing 0.5% FBS. The cells were starved for 24 h. Subsequently, the cells were treated with different concentrations of TAT-rhbFGF and rhbFGF for 48 h. The cell density was measured by adding 20 μl MTT (5 mg/ml) per well for 4 h. Finally, the medium was replaced with 100 μl DMSO, and the absorbance was detected at 570 nm after shaking for 10 min.

### The effect of TAT-rhbFGF on hypertrophic scars

**The preparation of TAT-rhbFGF gel.** First, 0.25 g carbomer was combined with 500 μl mannitol solution and allowed to swell in 45 ml deionized water for 24 hours. Then, we added 1 ml 1 mol/l PB to the Carbomer gel. Additionally, 0.015 g methylparaben and 0.005 g ethylparaben were used as antiseptic substances. We utilized triethanolamine to adjust the pH of the Carbomer gel to 7.0. The viscosity of the Carbomer gel increased with increased basicity. The Carbomer gel was then sterilized via standard autoclaving. Finally, we mixed the recombined protein with the Carbomer gel to the required concentration after sterilization and stored it in the freezer.

**The ability of TAT-rhbFGF to penetrate cells.** Human foreskin fibroblasts (HFFs) were used for the penetration trials. The cells (10×10^4^ per well) were seeded into a 6-well plate containing a sterilized microslide and DMEM. The microslide was dipped in 75% ethyl alcohol for 8 h on a clean bench and was washed with PBS before use. The DMEM contained 10% FBS, 100 U/ml ampicillin, 100 U/ml streptomycin, and 1.5 g/l glucose. After 12 hours, the cells had adhered to the microslide. We then treated the cells with TAT-rhbFGF and rhbFGF for 15 min with a final concentration of 15 ng/ml. Then, the microslides were washed 3 times in PBS and fixed for 15 min by previously frozen 4% paraformaldehyde at 4°C. Next, the cells were exposed to 0.3% triton-x-100 for 10 min. The proteins penetrating into the cells were detected using rabbit anti-human FGF-2 polyclonal antibodies followed by goat anti-rabbit fluorescent secondary antibodies.

**The ability of TAT-rhbFGF to penetrate the skin of a mouse.** BALB/c male mice (20–25 g, n = 15) were anesthetized with chloral hydrate (300 mg/kg, Sigma, Germany), and their dorsal hair was carefully shaved (3 cm×3 cm segment). The animals were divided into three groups (n = 5): a normal group, a control group and a treatment group. The treatment group was daubed with 200 μl TAT-rhbFGF gel containing 40 μg TAT-rhbFGF, and the control group was treated the same except the rhbFGF protein was utilized. The normal group was treated with a blank Carbomer gel. After 30 minutes, the mice were euthanasia by cervical dislocation. The skin segments were fixed and embedded in paraffin. Then, the skin samples were cut into 5 μm thick sections using standard procedures. Finally, the sections were subjected to immunohistochemical analysis.

**The effect of TAT-rhbFGF gel on hypertrophic scars in a rabbit ear model.** The rabbit ear model of hypertrophic scars was established as described previously with a minor modification[18]. Six Japanese big-ear white rabbits were anesthetized with sodium pentobarbital (30 mg/kg, Sigma, Germany) by intraperitoneal injection. Four identical, full-thickness, circular wounds with a 1 cm diameter were created down to the cartilage on the ventral surface of each ear using a surgical blade. The wounds were exposed to air and cleaned every day. Four weeks later, the hypertrophic scar model was established with a prominence in the central scar. Then, the hypertrophic scars were randomly divided into three groups (n = 8): control, TAT-rhbFGF, and rhbFGF and were treated with blank Carbomer gel, TAT-rhbFGF gel (6 μg), and rhbFGF gel (6 μg), respectively, for 30 days. Every ten days, two rabbits were sacrificed by air embolism. The scar tissue in every group was harvested and embedded in paraffin after being fixed in 4% paraformaldehyde. The hypertrophic scar was assessed in terms of thickness, fibroblast density, the content of collagen and cell apoptosis. The thickness of hypertrophic scars is the ratio of the central thickness of the model group to the normal group. All measurements were performed within the confines of the scar using 40 × magnification of the H&E stained tissue sections. The fibroblast density was measured by 6 random microscopic inspections of the H&E stained slices at 400×. To determine of the number of fibroblasts in a unit area and the average fibroblast densities for the entire group, the ratios between the cell numbers of the model group and those of the normal group were computed. The transition of collagen content in the scars was detected by Masson staining. The stained slices were randomly selected for microscopic examination at 200×. The One Step TUNEL Apoptosis Assay Kit was used to detect cell apoptosis and was implemented in accordance with the manufacturer’s protocol.

## Results

### Construction of TAT-rhbFGF expression vector

**Fig 1:**
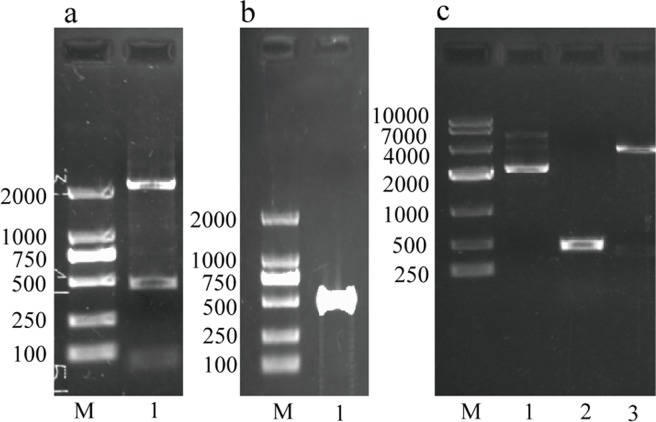
Agarose gel electrophoresis results from the identified recombinant gene. In section a, lane 1 is the product of the first PCR step; in section b, lane 1 is the final recombinant gene; in section c, lane 1 is the pET3c plasmid vector, lane 2 is the PCR product of the transformant, and lane 3 is the digested recombinant vector product.

To acquire the TAT-rhbFGF coding sequence, we amplified the gene as described in the methods section. The first and second PCR products are shown in Fig. 1(A, B). Then, we digested the second PCR product and connected it to the pet3c expression vector. We abstracted and digested the plasmid, which formed the DH5α E. coli containing target sequence. Additionally, we detected the gene sequence by bacterium polymerase chain reaction (Fig. 1C). The lengths of all the products were in accordance with their theoretical value. The TAT-rhbFGF sequence was confirmed by automated DNA sequencing.

### Production and screening of TAT-rhbFGF

**Fig 2:**
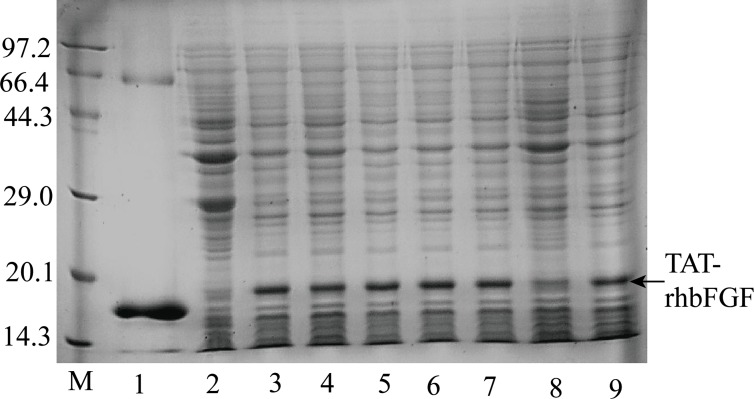
The analysis of TAT-rhbFGF expression using SDS-PAGE. Lane M is a MW marker (low), lane 1 is the rhbFGF control, lane 2 is the expression of the BL21 (DE3)pLsS cells without the inducer, and lanes 3 through 6 are the expression of BL21 (DE3)pLsS cells after being induced for 1 to 4 hours.

To obtain the TAT-rhbFGF protein, we utilized BL21 (DE3) pLsS bacteria for an expression screening and performed several small-scale experiments. The main protein was highly produced in the BL21 (DE3) pLsS cells, as verified by SDS-PAGE and shown in Fig. 2. For this work, we harvested the highly expressed cells and stored them in a freezer at -70°C.

### Purification and identification of TAT-rhbFGF

**Fig 3:**
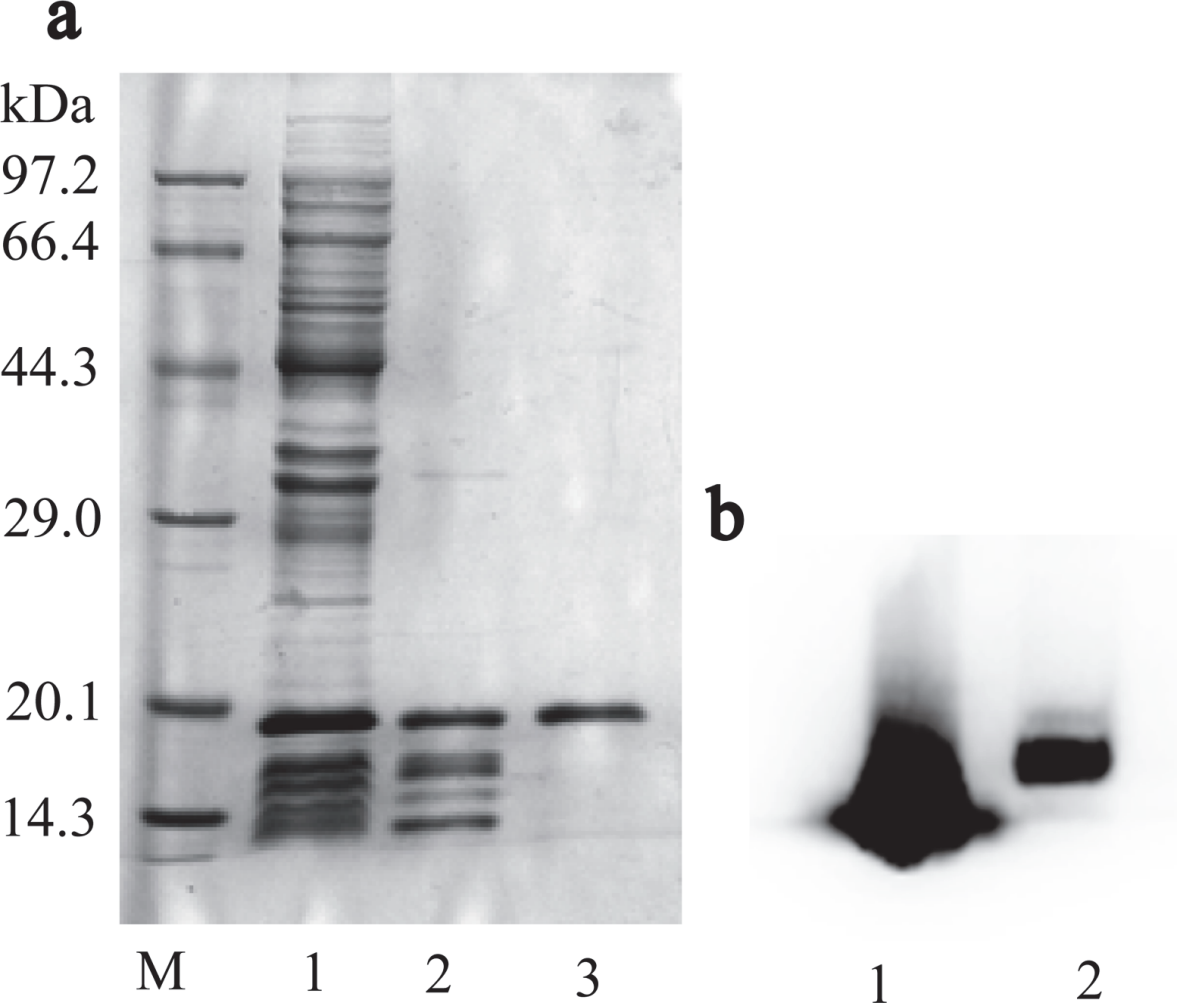
The purification and identification of TAT-rhbFGF. In section a, lane 1 is the supernatant of the cell lysate, lane 2 is the purified product from the CM Sepharose, and lane 3 is the final product of the heparin sepharose chromatography. In section b, lane 1 and lane 2 are the western blot results for rhbFGF and TAT-rhbFGF, respectively.

The purification of TAT-rhbFGF was conducted as described in the “materials and methods” section. The supernatant of the cell lysate was applied to CM sepharose, and quantities of the mixed proteins were discarded. Then, the protein was purified by a heparin sepharose column, and the protein purity was confirmed to be greater than 95%. As shown in the Fig. 3(A), the final purified product was almost a single band tested by SDS-PAGE. We identified TAT-rhbFGF via western blot (Fig. 3B), in which the protein was transformed to a nitrocellulose membrane and detected using the bFGF antibody.

### Analysis of the mitogenic activity of TAT-rhbFGF

**Fig 4:**
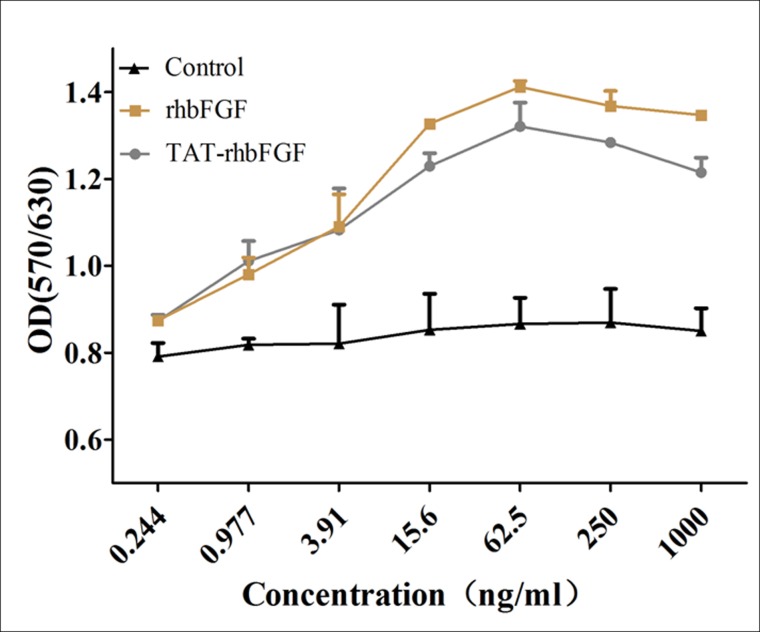
The proliferation activity of TAT-rhbFGF was tested using NIH 3T3 Cells. NIH3T3 cells, which were seeded into 96-well microplates at 7000 cells/well, were allowed to attach (4–6 hours) and were then incubated in DMEM with 0.5% fetal bovine serum overnight at 37°C. Cells were supplemented with rhbFGF and TAT-rhbFGF and were incubated at 37°C for 48 hours. PBS was used as a control.

In the MTT assay, the bioactivity of TAT-rhbFGF was similar to the mitogenic activity of rhbFGF. The bioactivity of TAT-rhbFGF was slightly greater than that of rhbFGF at very low concentrations; however, the bioactivity was slightly less than that of rhbFGF when the protein concentration was greater, as shown in Fig. 4.

### The ability of TAT-rhbFGF to penetrate cells

**Fig 5:**
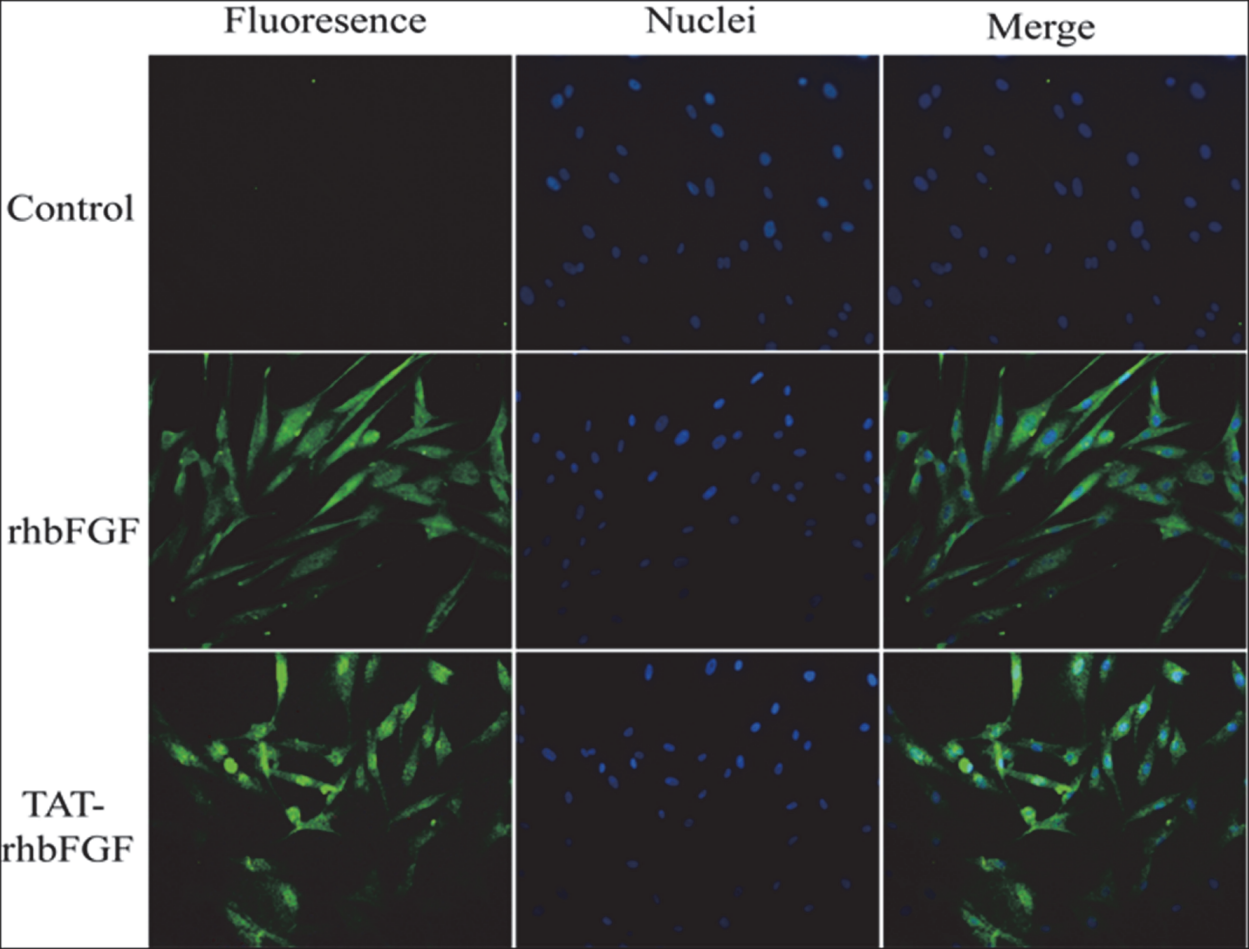
The ability of TAT-rhbFGF to penetrate cells. These images were produced using immunofluorescence. The target protein was identified by green fluorescence.

To determine whether TAT-rhbFGF has the ability to penetrate fibroblasts, we utilized the human foreskin fibroblasts. The detailed processing was conducted in accordance with the “materials and methods” section. The proteins were detected using cell immunofluorescence, and the result is shown in Fig. 5. In the figure, we can see that both TAT-rhbFGF and rhbFGF could penetrate into cells. However, the amount of TAT-rhbFGF penetrating into the cells was much greater than that of rhbFGF.

### The ability of TAT-rhbFGF to penetrate the skin of a mouse

**Fig 6:**
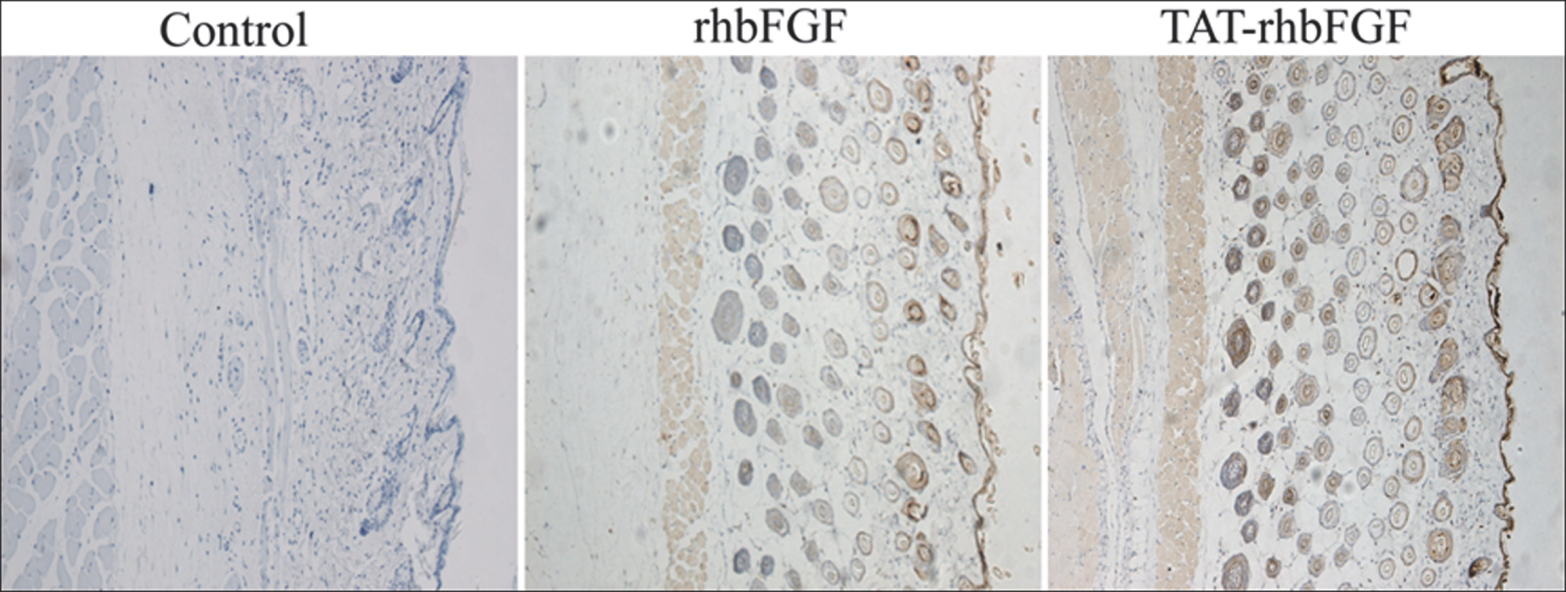
TAT-rhbFGF and rhbFGF penetrated into the skin of a mouse. The nuclei stained blue, and the positive proteins stained brown.

In this trial, the skins were embedded in paraffin and detected via immunohistochemistry. As shown in Fig. 6, rhbFGF primarily aggregated in the hair follicle and in the surface of the mouse skin. However, TAT-rhbFGF could not only aggregate in the subcutaneous hair follicle but could also directly penetrate into the dermal tissue through the skin barrier. In this study, we can conclude that the subcutaneous penetration of TAT-rhbFGF was readily available and was absorbed faster than rhbFGF.

### Analysis of the effect of TAT-rhbFGF gel on hypertrophic scars in the rabbit ear model

The physical appearance of the hypertrophic scars gradually turned to a pale red after treatment for 30 days. Compared with the control group, the protuberant scars became flat in the TAT-rhbFGF groups. The thickness of the scar tissue is shown in Table 1. From the data, we concluded that TAT-rhbFGF improved the hypertrophic scars by reducing the thickness and the overall appearance.

**Table 1:** Effect of TAT-rhbFGF on the index of hypertrophic scar thickness.

group	n	10 days after therapy	20 days after therapy	30 days after therapy
control	8	2.79±0.10	2.75±0.11	2.58±0.13
rhbFGF	8	2.80±0.11	2.54±0.12*	2.46±0.13*
TAT-rhbFGF	8	2.77±0.12*	2.05±0.17*	1.61±0.10*

Note: Data are presented as the mean±SD.

* Compared with the control, *P*<0.05.

**Fig 7:**
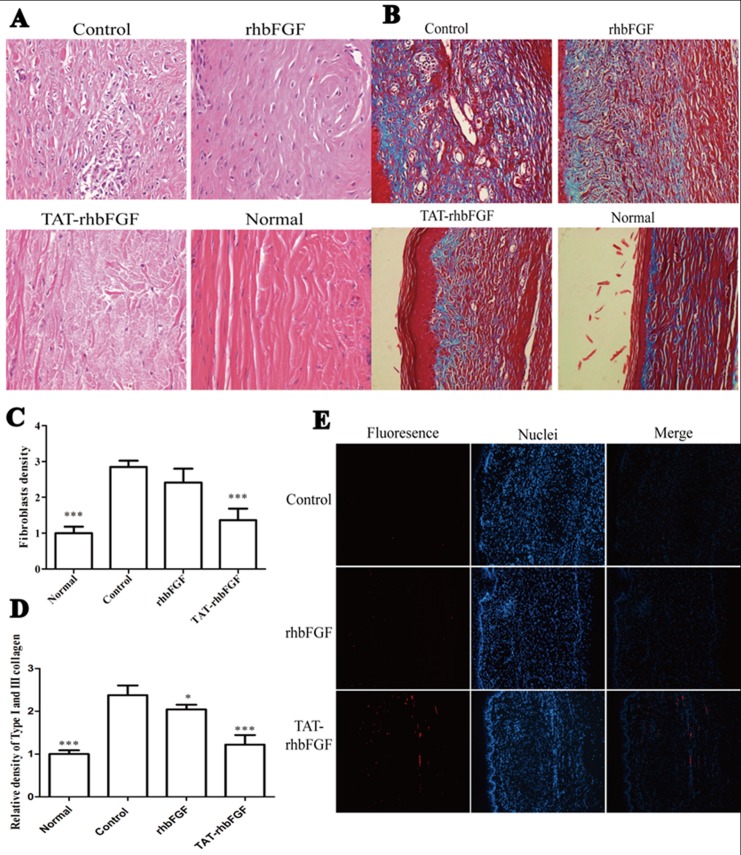
The effect of rhbFGF and TAT-rhbFGF gel on the hypertrophic scar model. (A and C): the tissue slices of the hypertrophic scar stained using H&E, A: observed at 400×, C: the relative density of fibroblasts; all the data are compared with the mean density of the normal derma. * Compared with the control, *P*<0.05; (B and D): the content of type I and III collagen in the scars, B: the collagen stained using a Masson kit, observed at 200× D: the relative content of type I and III collagen; all the data are compared with the mean content of the normal derma. *P<0.05 and ***P<0.01 compared with the control; (E): the apoptosis cells in HTS after treatment observed at 200×.

The cell density of the TAT-rhbFGF treatment group was much lower than that of the rhbFGF group and the control group, and there was a similar cell density compared with the normal group (Fig. 7A). In the scar tissue, there was greater collagen accumulation, and the distribution of collagen was uneven compared with the normal group (Fig. 7B). After topical application of TAT-rhbFGF for 30 days, type I and III collagen in the scar tissue were reduced and evenly distributed. From the results of the TUNEL staining, we found that the number of apoptotic cells in the TAT-rhbFGF group was greater compared with the control and rhbFGF groups. Apoptotic cells were absent in the control group (showed in Fig. 7 E).

## Discussion

rhbFGF has been shown to be a powerful wound healing factor, and its effect on mature scars has yet to be researched extensively[12]. To improve rhbFGF’s penetration ability, we utilized the TAT peptide as a transport vessel in this study. Expression of the exogenous protein in Escherichia coli is a technique that has being used more in recent years due to its success rate and cost effectiveness[19–21]. The pET3c vector is a frequently used vector that adopts the T7 RNA polymerase to selectively activate the T7 phage promoter in E. coli. The T7 RNA polymerase that is inserted into the pET3c vector is transient and inducible during the expression of exogenous proteins[22]. Considering that the bioactivity of rhbFGF may be inhibited when a fusion protein attaches to its C terminal[23], we fused the TAT sequence to rhbFGF’s N terminal. Therefore, we acquired a pET3c vector containing the TAT-rhbFGF encoding sequence.

The recombinant TAT-rhbFGF protein was purified via three purification columns, which included CM Sepharose FF, heparin sepharose column and Sephadex G-25. These columns have been broadly and successfully used for the purification of rhbFGF and TAT-rhbFGF[24]. The purity of this recombinant protein was greater than 95%, as detected by SDS-PAGE, and was identified by western blot. The bioactivity of TAT-rhbFGF was verified by stimulating the proliferation of NIH-3T3 cells, which demonstrated that the TAT peptide may assist rhbFGF penetration into the cell and deposition to the receptor location. Furthermore, our cell penetration trial combined with the bioactivity trial demonstrated that the TAT peptide may cause nuclear localization of the fusion protein[25,26]. Previous studies showed that some biological activities of bFGF may be mediated by the direct binding of bFGF to DNA, which supports the effect of the TAT peptide in its role to assist in cell penetration[27]. In the transdermal trial, the fusion protein could penetrate into the skin as described in previous studies[28,29].

Hypertrophic scars are formed through a complicated and multifactorial participation process[30]. Many scientists have attempted to resolve the intractable symptom through the development of pressure therapy, corticosteroids, laser therapy, cryotherapy and even surgery for the treatment of exuberant scars[31–33]. Recently, rhbFGF was confirmed to prevent and alleviate hypertrophic scar formation in a rabbit hypertrophic scar model[34]. The aberrant proliferation of fibroblasts is seen as an important inducing factor of hypertrophic scars not only in inducing the scar to become hypertrophic but also in the excessive deposition of ECM. The ECM products primarily consist of type I and III collagen. Although type I and III collagen are indispensable components for wound healing, they can lead to scarring with excessive expression[35,36]. TAT-rhbFGF was able to reduce the incidence of type I and III collagen, which may lead to an increase in scar formation. To have a further understand of how the scar was improved after the application of TAT-rhbFGF, we detected the proliferation of fibroblasts in the scar. The TUNEL trial indicated that the effect of the TAT-rhbFGF on improving the scar was linked to the apoptosis of fibroblasts.

In summary, we have successfully expressed and purified a TAT-rhbFGF fusion protein in this study. Our results demonstrated that the fusion protein had better penetration to the dermal areas of the skin. Additionally, TAT-rhbFGF improved the physical appearance of the hypertrophic scars. TAT-rhbFGF may be a potential fusion protein for the treatment of dermal disorders including hypertrophic scars.
